# Supplementary material for: Joining of metallic glasses in liquid via ultrasonic vibrations
Source: Nat Commun. 2023 Oct 9;14:6305. doi: 10.1038/s41467-023-42014-x (PMC10562460; doi:10.1038/s41467-023-42014-x)
Supplement: Supplementary file 1 — Supplementary Information [file 41467_2023_42014_MOESM1_ESM.pdf]

Supplementary Information for

Joining of metallic glasses in liquid via ultrasonic vibrations

Luyao Li[1], Xin Li[2], Zhiyuan Huang[3,4], Jinbiao Huang[1], Zehang Liu[1], Jianan Fu[5], Wenxin Wen[1], Yu Zhang[1], Shike Huang[1], Shuai Ren[1], Jiang Ma[1*]

[1]Shenzhen Key Laboratory of High Performance Nontraditional Manufacturing, College of Mechatronics and Control Engineering, Shenzhen University, Shenzhen, 518060, China.

[2]School of Mechanical, Electrical and Information Engineering, Shandong University, Weihai 264209, China

[3]Songshan Lake Materials Laboratory, Dongguan 523808, China

[4]School of Materials Science and Engineering, Shanghai University, Shanghai 200444, China

[5]Department of Mechanics and Aerospace Engineering, Southern University of Science and Technology, Shenzhen 518055, China

[*] To whom correspondence should be addressed: majiang@szu.edu.cn

This file includes:

Supplementary Figure 1. The sample display chart.

Supplementary Figure 2. The temperature-time curve.

Supplementary Figure 3. The joining performance of heterogeneous joined samples.

Supplementary Figure 4. The observation of dispersed oxide layer under TEM.

Supplementary Figure 5. The tensile fracture surface morphology.

Supplementary Figure 6. The corrosion morphology observation of heterogeneous joined samples.

Supplementary Figure 7. The EDS mapping analysis under SEM.

Supplementary Figure 8. The equipment diagrams.

Supplementary Figure 9. The clamping method diagram.

Supplementary Figure 10. The details of temperature measurement.

Supplementary Figure 11. The process diagram of tensile sample preparation.

Supplementary Figure 12. The joined specimen used for corrosion testing.

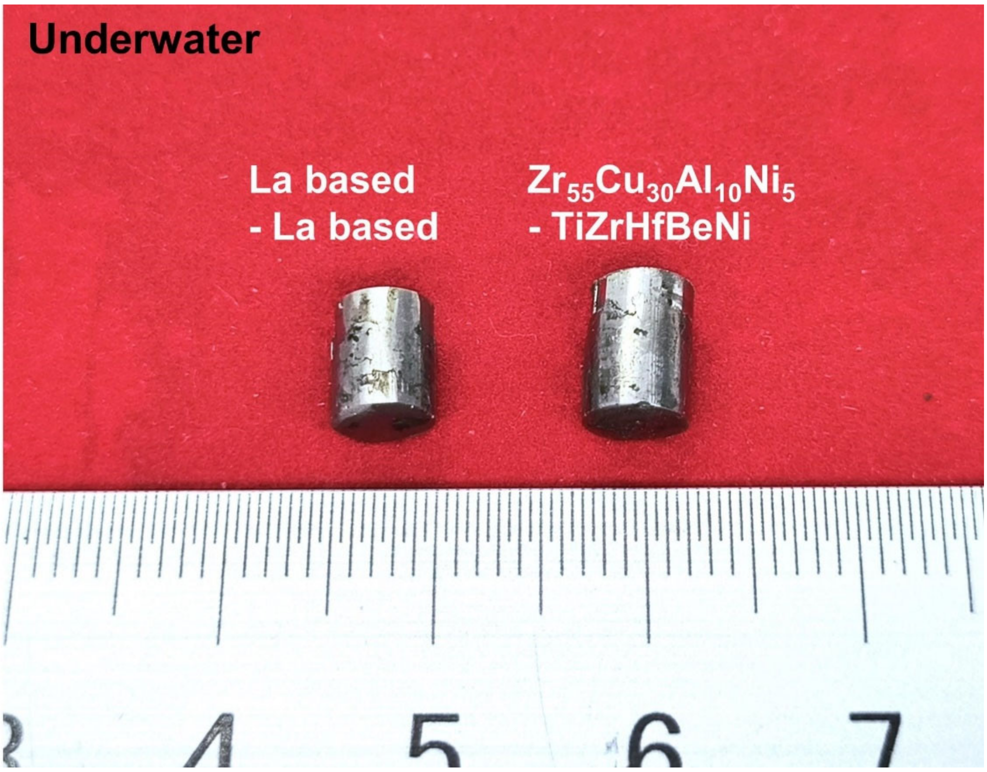

Supplementary Figure 1. The sample display chart of La-based ($La_{55}Al_{25}Ni_{5}Cu_{10}Co_{5}$) joined sample and heterogeneous ($Zr_{55}Cu_{30}Al_{10}Ni_{5}$-TiZrHfBeNi) joined sample.

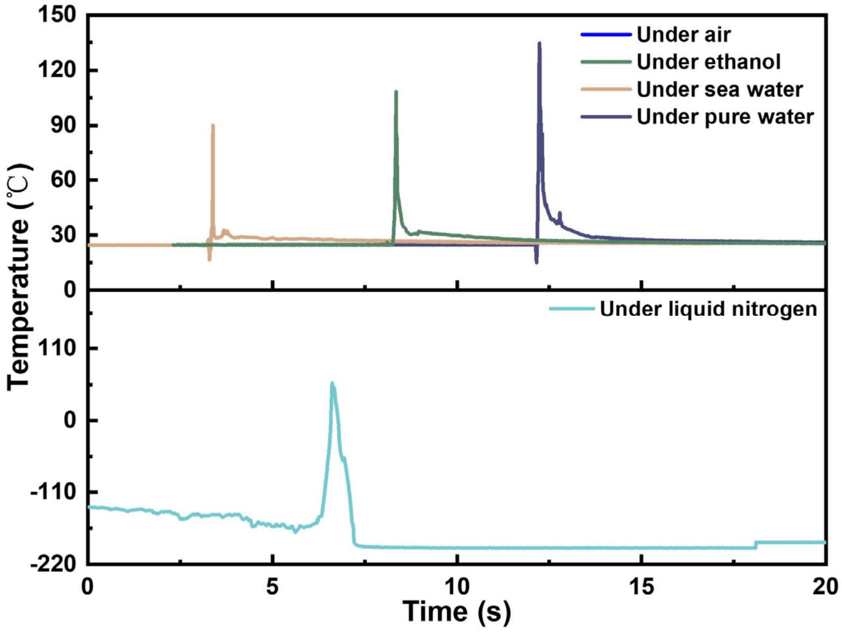

Supplementary Figure 2. The temperature-time curve of the La-based joining process is measured by the thermocouple.

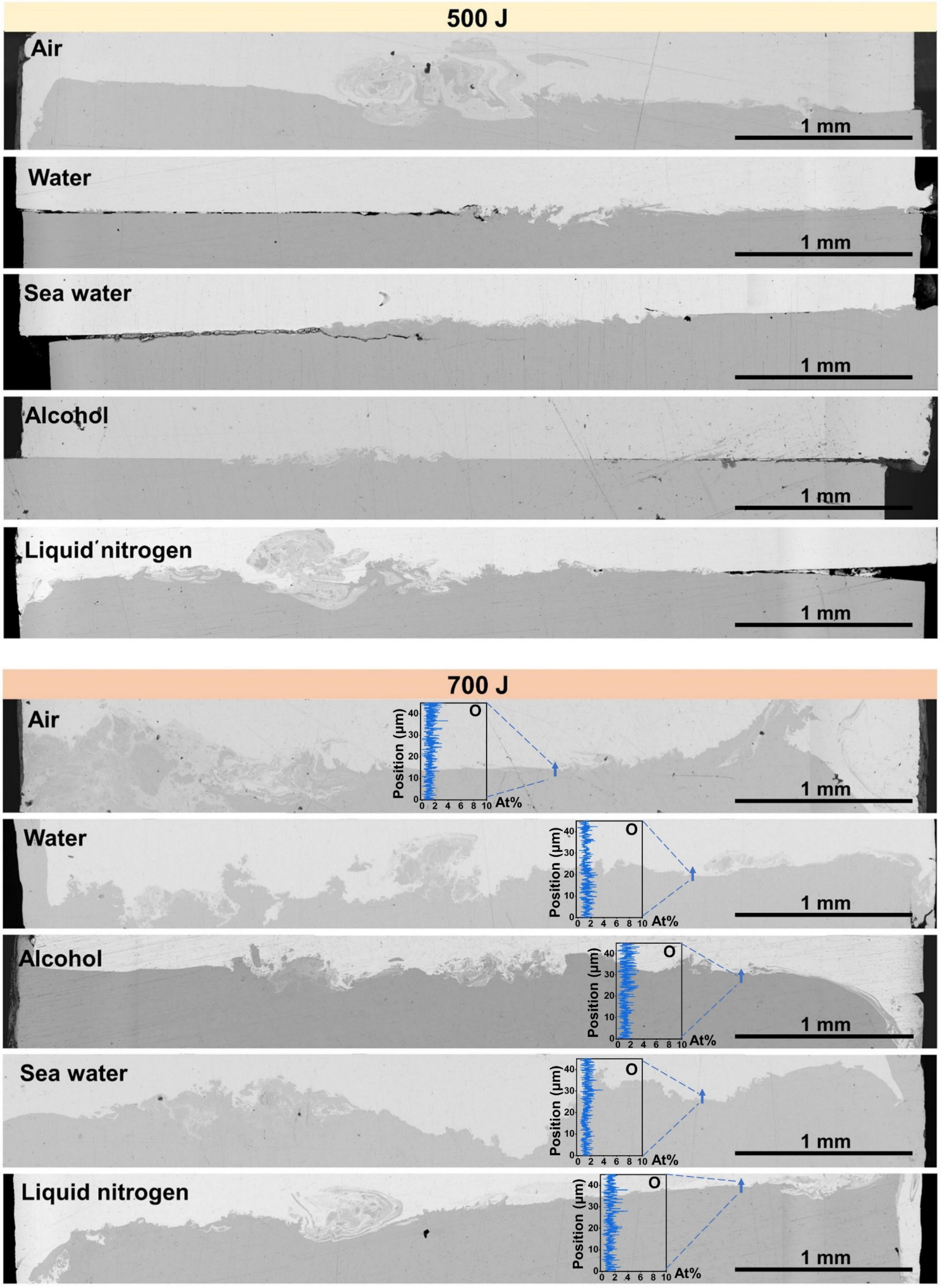

Supplementary Figure 3. The joining performance of heterogeneous joined samples in air and different liquids (pure water, seawater, alcohol and liquid nitrogen). The inset shows the line scan of the oxygen element at the interface.

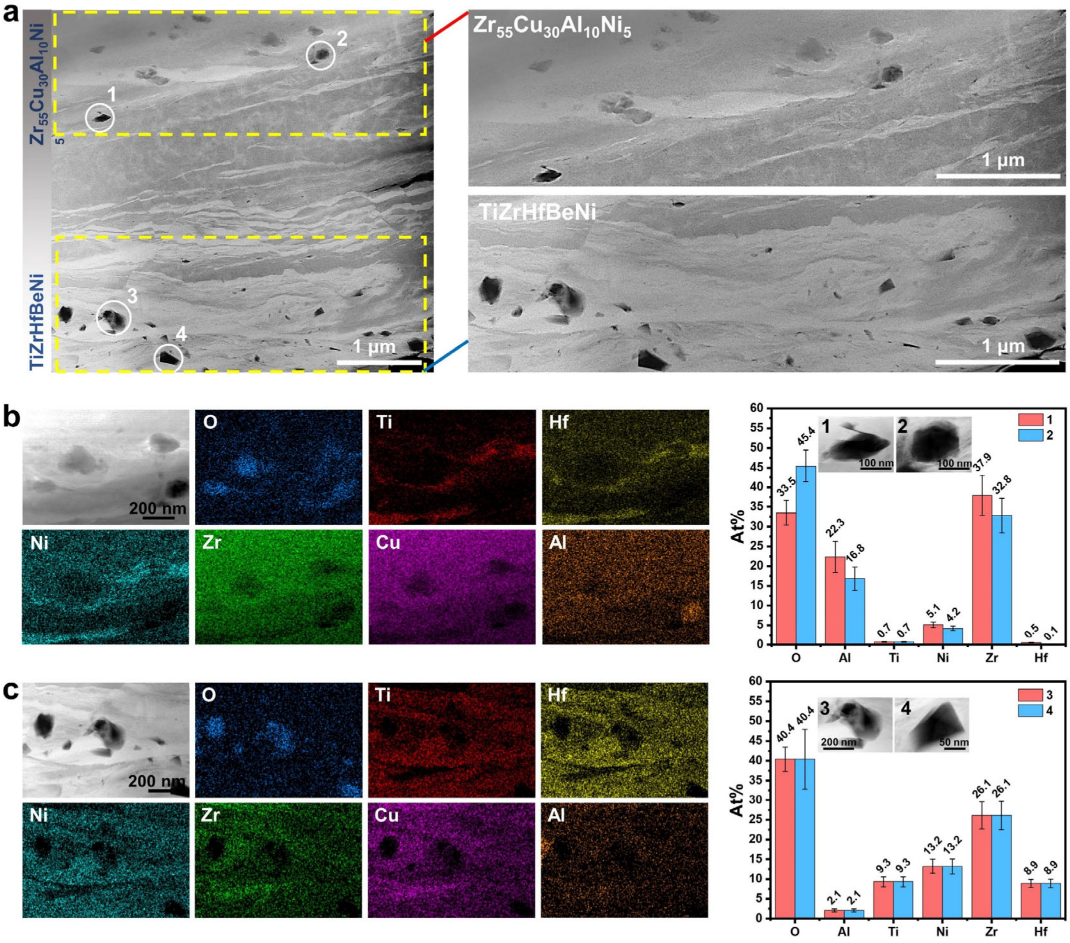

Supplementary Figure 4. The observation of dispersed oxide layer under TEM. a The high angle annular dark field (HAADF) images at the interface of the heterogeneous joined sample, which show dispersed oxide particles and mixed interfaces. **b** Elemental analysis of $Zr_{55}Cu_{30}Al_{10}Ni_5$ MG oxide particles. **c** Elemental analysis of TiZrHfBeNi MG oxide particles. The error bars represent the systematic relative errors of elemental content obtained from the nanobeam EDX measurement.

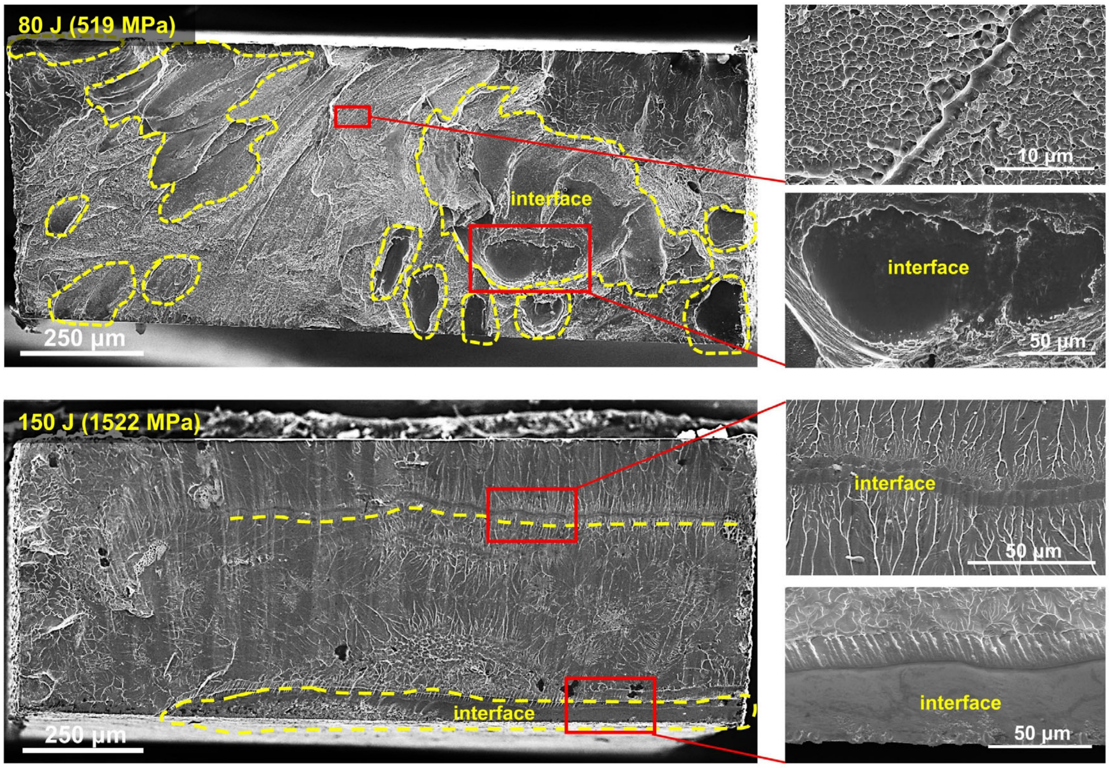

Supplementary Figure 5. The tensile fracture surface morphology of Zr-based joined sample at different energies. Due to the use of butt joint (sheet) samples in the tensile tests, the overall value of welding energy applied is relatively small.

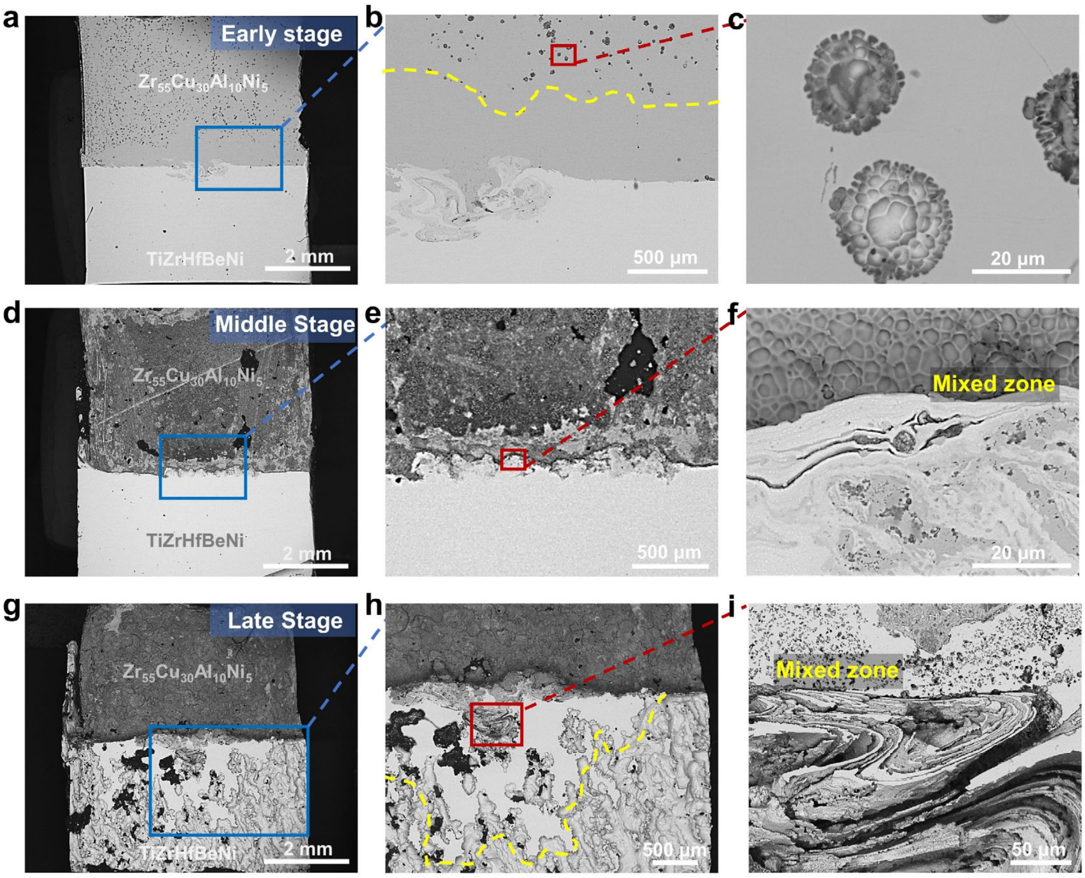

Supplementary Figure 6. The corrosion morphology observation of heterogeneous joined samples. a-c The field emission scanning electron microscope (SEM) images of the early stages in corrosion testing, which show no significant pitting near the interface. **d-f** The SEM images of the middle stages in corrosion testing. The results show that one side of the $Zr_{55}Cu_{30}Al_{10}Ni_5$ phase was fully corroded, but the two-phase mixed zone still contained the remaining $Zr_{55}Cu_{30}Al_{10}Ni_5$ phase. **g-i** The SEM images of the late stages in corrosion testing. The results show that the TiZrHfBeNi phase near the interface was not corroded and the $Zr_{55}Cu_{30}Al_{10}Ni_5$ phase in the mixed region was completely corroded.

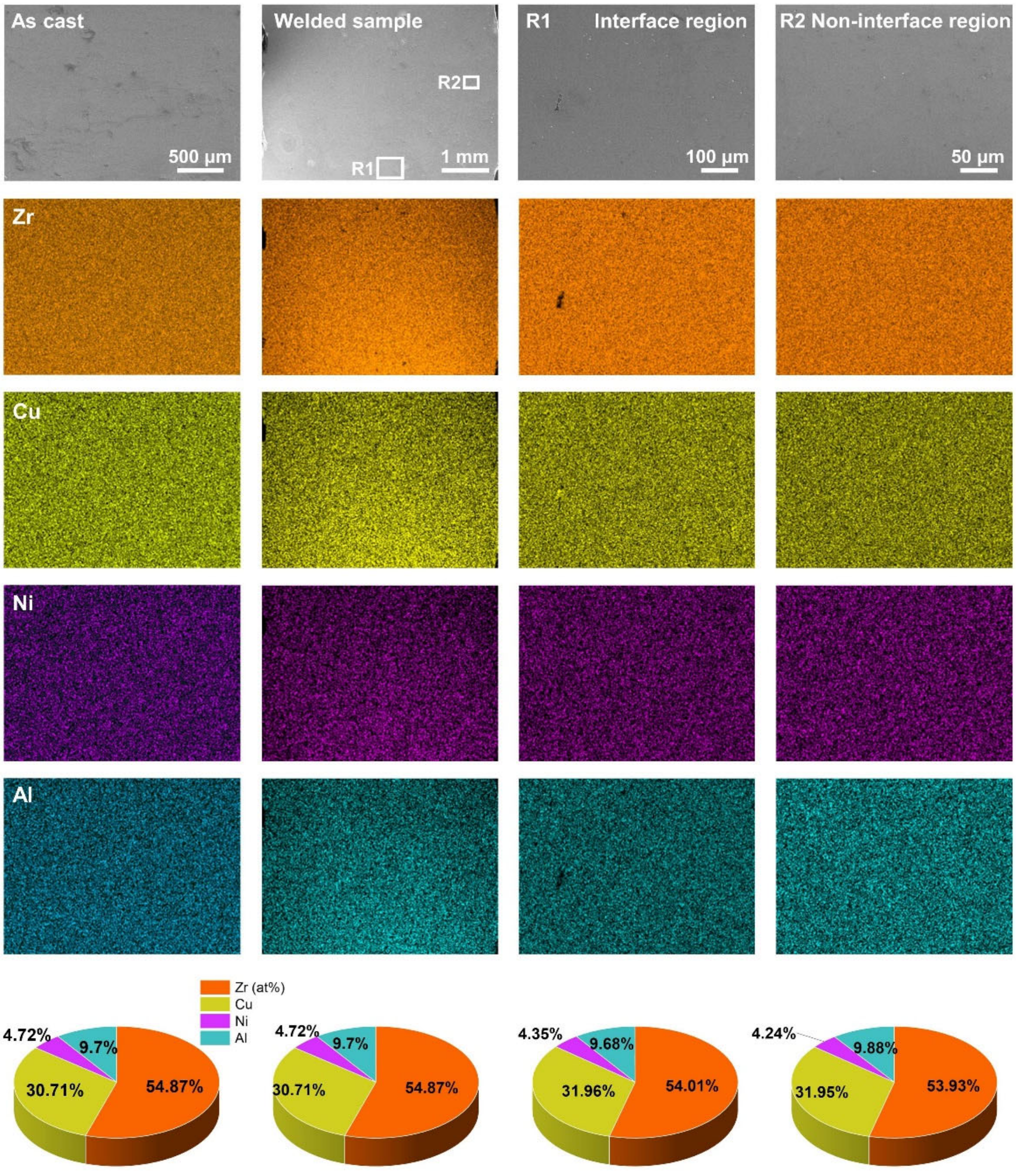

Supplementary Figure 7. The EDS mapping analysis under SEM of as cast Zr-based MG and joined sample cross-sections.

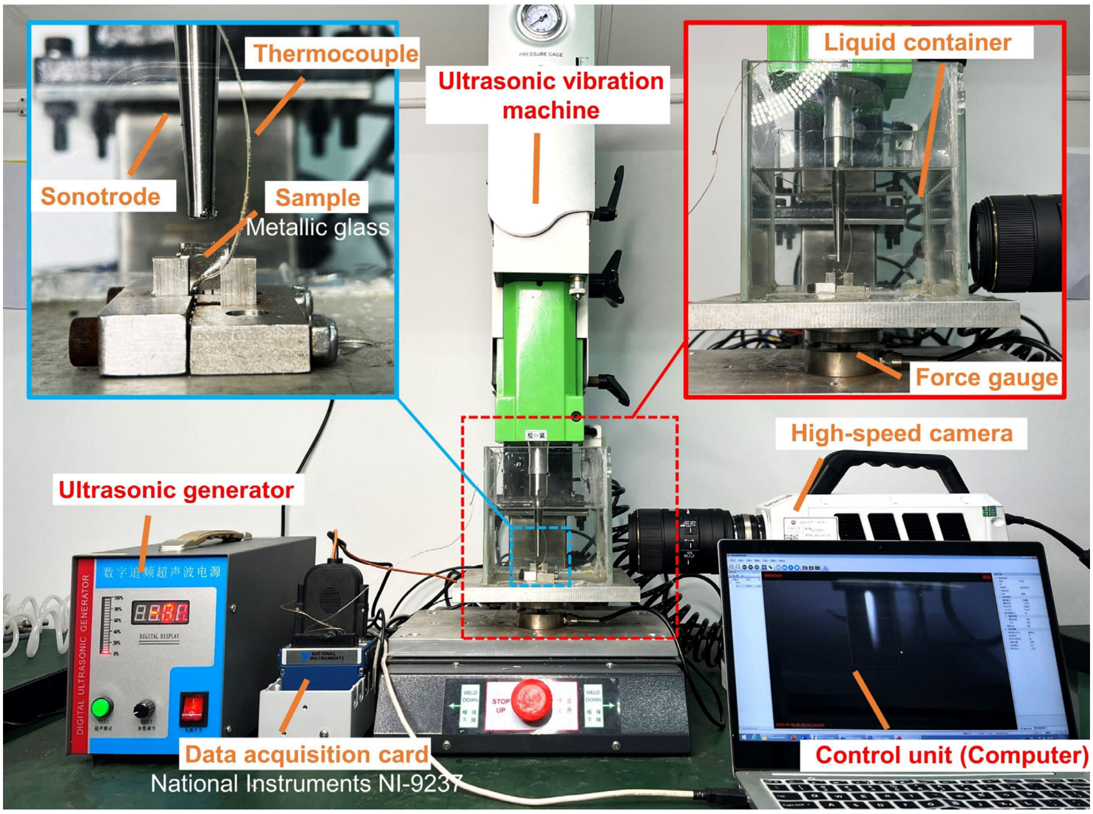

Supplementary Figure 8. The equipment diagrams.

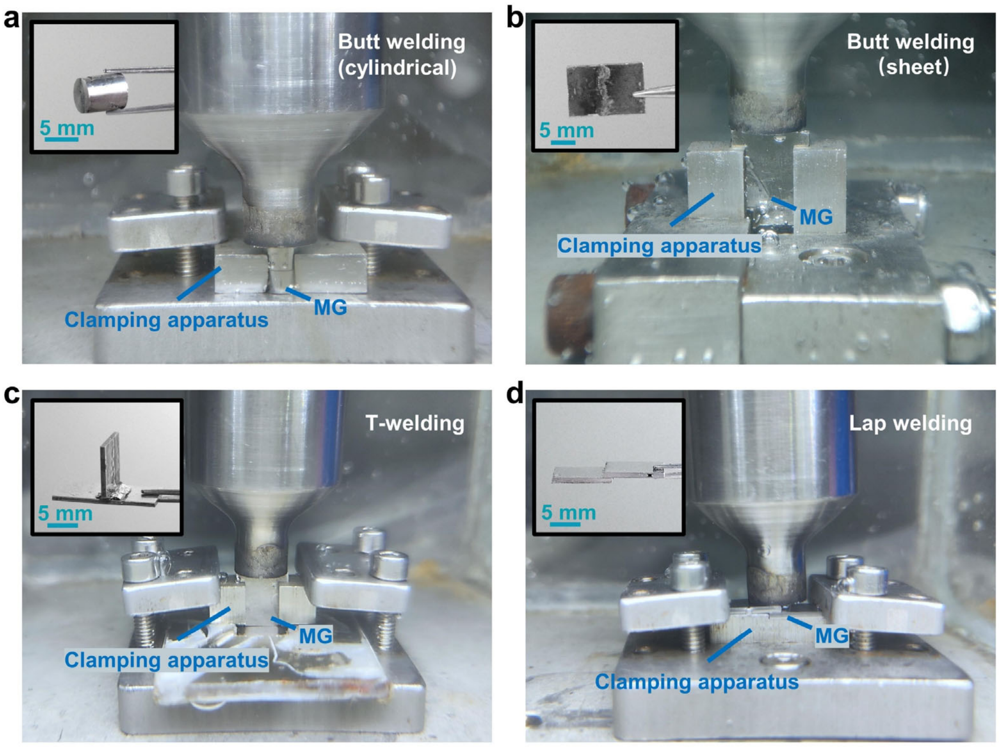

Supplementary Figure 9. The clamping method diagram of 4 different types of joining. **a** Butt joint of cylindrical. **b** Butt joint of sheet. **c** Lap joint. **d** T-joint.

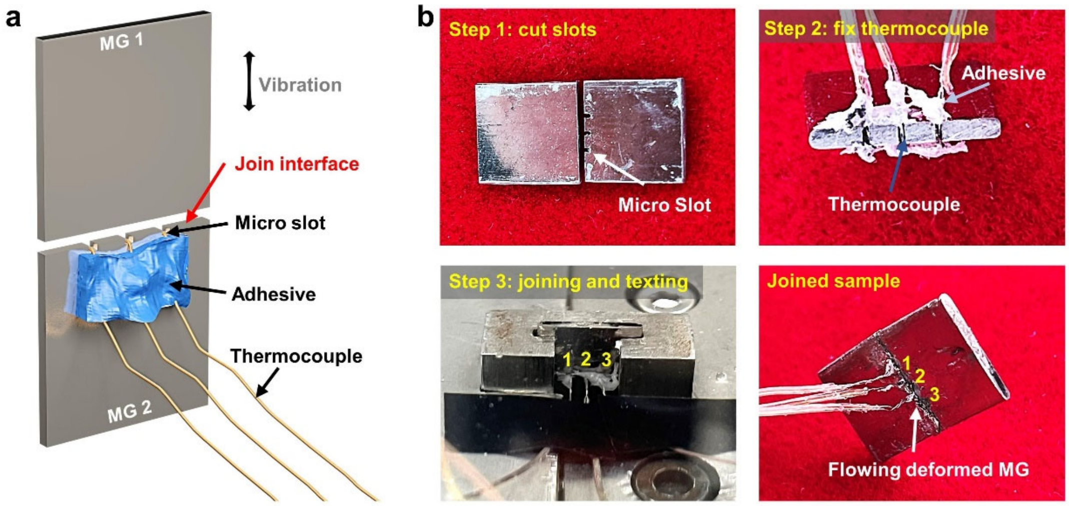

Supplementary Figure 10. The details of temperature measurement. a Schematic diagram of the temperature measurement method. **b** Physical display of the temperature measurement process.

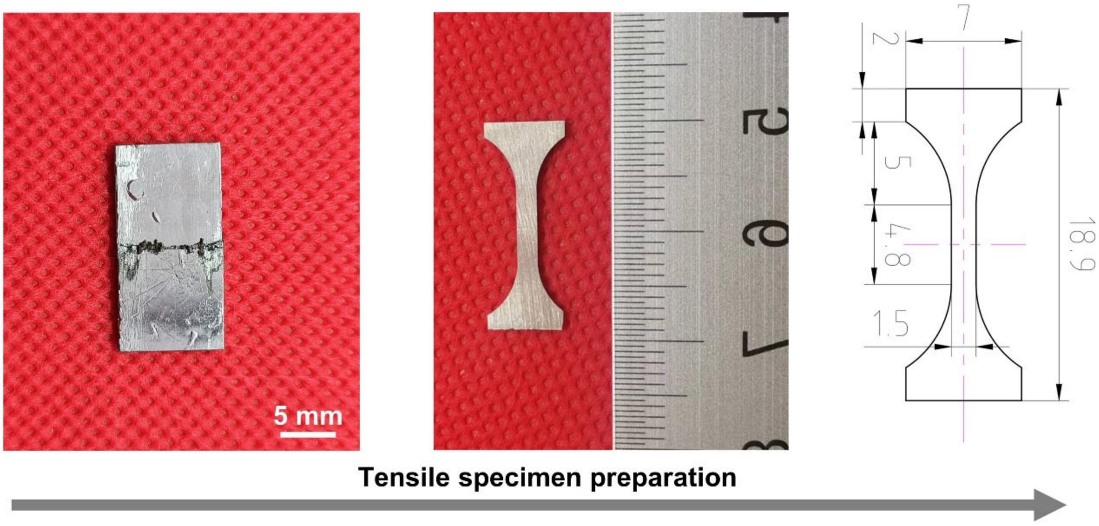

Supplementary Figure 11. The process diagram of tensile sample preparation.

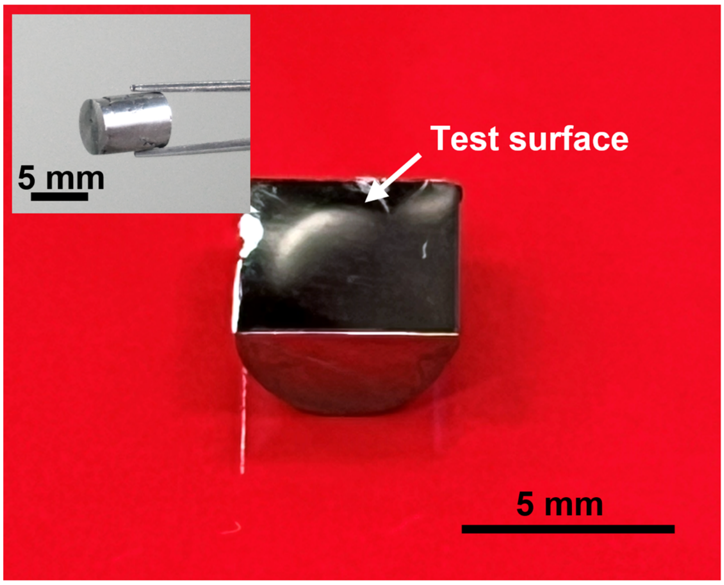

Supplementary Figure 12. The joined specimen used for corrosion testing.
