# Supplementary material for: Joining of metallic glasses in liquid via ultrasonic vibrations
Source: Nat Commun. 2023 Oct 9;14:6305. doi: 10.1038/s41467-023-42014-x (PMC10562460; doi:10.1038/s41467-023-42014-x)
Supplement: Supplementary file 3 — Description of Additional Supplementary Files [file 41467_2023_42014_MOESM3_ESM.pdf]

Description of Additional Supplementary Files

File Name: Supplementary Movie 1
Description: The slow play process of joining Zr-based MG ($Zr_{55}Cu_{30}Al_{10}Ni_5$) in pure water.

File Name: Supplementary Movie 2
Description: The slow play process of joining Zr-based MG ($Zr_{55}Cu_{30}Al_{10}Ni_5$) in sea water.

File Name: Supplementary Movie 3
Description: The slow play process of joining Zr-based MG ($Zr_{55}Cu_{30}Al_{10}Ni_5$) in ethanol.

File Name: Supplementary Movie 4
Description: The slow play process of joining La-based MG ($La_{55}Al_{25}Ni_5Cu_{10}Co_5$) in pure water.

File Name: Supplementary Movie 5
Description: The slow play process of joining La-based MG ($La_{55}Al_{25}Ni_5Cu_{10}Co_5$) in sea water.

File Name: Supplementary Movie 6
Description: The slow play process of joining La-based MG ($La_{55}Al_{25}Ni_5Cu_{10}Co_5$) in ethanol.

File Name: Supplementary Movie 7
Description: The joining procedure in liquid nitrogen captured by the normal camera.
